# Emotionally based strategic communications as a new tool in defensive cognitive warfare

**DOI:** 10.3389/fpsyg.2026.1751406

**Published:** 2026-02-06

**Authors:** Krešimir Ćosić, Siniša Popović, Brenda Wiederhold

**Affiliations:** 1 University of Zagreb Faculty of Electrical Engineering and Computing, Zagreb, Croatia; 2 Virtual Reality Medical Center, San Diego, CA, United States

**Keywords:** artificial intelligence, cognitive warfare, cognitive neuroscience, emotionally based strategic communications, European defense and security, large language model

## Abstract

In modern cognitive warfare, adversaries deliberately target human cognition, emotion, belief, trust, and decision-making processes, seeking to destabilize democratic societies through disinformation and divergent media campaigns. This article argues that the growing accessibility and vulnerability of the human emotional brain to external influence in a technologically connected world has important repercussions for global defense and security strategy. Recent EU/NATO strategic documents emphasize the need to strengthen resilience, counter hybrid/cognitive threats, and protect societies against disinformation and manipulation. Resilience to cognitive warfare, however, depends on distributed societal capacities for emotional literacy, deliberation, and comprehension—grounded in emotional and cognitive superiority, political culture, robust democratic institutions, and an informed public. Deep security crises and prolonged military conflicts arouse strong negative emotions among affected individuals, groups, and societies. Accordingly, this article proposes Emotionally Based Strategic Communications (EBSC) as a scientifically and ethically grounded approach for applying cognitive neuroscience and artificial intelligence (AI) to design emotionally resonant, legitimate, and strategically aligned communications, aiming to strengthen societal cohesion, counter adversary narratives, and build societal resilience against cognitive threats. EBSC provides tools for identifying and transforming dominant emotional states within target populations through the intentional design of structured multimodal narratives, language, imagery, and symbolic framing, with the aim of positively reconfiguring collective emotions without coercion. EBSC is conceptualized as a Large Language Model (LLM)–based systematic approach to strategic communications, which senses the emotional climate of target populations via social-sentiment analysis algorithms applied to various open digital sources; interprets and contextualizes this emotional climate; conducts design and development of appropriate output messages; delivers these messages across mass media; assesses their impact; and adapts them in a real-time closed loop, under supervision of accountable human decision-makers. The article calls for integrating the proposed closed-loop, LLM-based EBSC approach into the European defense ecosystem and strategic communications policy, aligned with EU frameworks on resilience and counter-disinformation. Such integration may offer a means of bridging cognitive neuroscience and AI into operational, scientifically informed, and emotionally resonant strategic communications that counter adversary narratives, prepare the public to resist disinformation and psychological pressure, and strengthen trust, cohesion, and overall societal resilience among EU/NATO allies.

## 1 Introduction

A comprehensive analysis of negative emotions—their distribution, structure, and conditioning at the state level—can provide deeper insight into the character of potential cognitive warfare and the risk of military aggression. A clearer understanding of defense and security challenges, as well as the complex, turbulent, and uncertain political environments surrounding them, can be achieved through mapping dominant emotional patterns that illustrate the mental and emotional states of affected populations. Therefore, emotion analysis should play an increasingly important role in international political and security analysis.

Augmenting political and security analyses with emotional considerations complements and enhances traditional rationality-based approaches, shifting from a purely rational framework toward a more integrative emotional-cognitive methodology (McDermott, 2004; Mercer, 2010; Crawford, 2014; Hall and Ross, 2015; Lerner et al., 2015). Long-lasting wars, severe security crises, and deep military conflicts arouse strong negative emotions among directly affected individuals, groups, and societies (Crawford, 2000; Bar-Tal, 2001; Bar-Tal et al., 2007; Jarymowicz and Bar-Tal, 2006; Lindner, 2009). Without understanding the influence of emotions—which shape the behavior of war-affected societies far more than these societies can control them—it is impossible to understand the political, psychological, and emotional aspects of any conflict.

For example, the protracted war in Ukraine is characterized by daily fighting, shelling, bombing, drone attacks, property destruction, violations of civil infrastructure, and inflammatory narratives. These conditions strongly affect the emotional experiences of the local population, generating a dramatic socio-political and emotional context (Limaj et al., 2024; Scharbert et al., 2024).

Today, unlike conventional kinetic warfare, cognitive warfare intentionally targets human cognition and performance—specifically, belief systems, social trust, collective attitudes, and political and institutional decision-making. The influence of cognitive warfare will be further empowered by advances in cognitive neuroscience and artificial intelligence (AI). This article argues that understanding the human emotional brain as increasingly vulnerable to external influence in a technologically interconnected world fundamentally reshapes contemporary defense and security strategy.

Cognitive warfare leverages psychological manipulation, disinformation, memetic engineering, and algorithmic influence to disrupt coherent sense-making within societies (du Cluzel, 2021; Claverie and du Cluzel, 2022; Deppe and Schaal, 2024; Marjanović and Smiljanić, 2025). Adversaries exploit affective vulnerabilities by engineering doubt, inducing moral fatigue, and triggering emotionally charged divisions across cultural, national, and political lines. The goal is not military destruction, but erosion of legitimacy, paralysis of decision-making, and weakening of social cohesion through AI-driven mechanisms.

Political leaders may underestimate the complexity and persistence of these emotional and psychological dynamics. Strong negative emotions—fear, humiliation, anger, hatred—can entrench group mentalities and significantly delay conflict resolution. Research in political psychology shows that negative emotional framing strongly influences public attitudes toward war, refugees, minority rights, and authoritarianism (Marcus et al., 2000; Lodge and Taber, 2013).

Emotionally Based Strategic Communications (EBSC; Cosić et al., 2011, 2012a,b, 2013, 2018), as proposed in this article, offers a structured method for applying cognitive neuroscience and AI within emotionally resonant, legitimate, and strategically aligned communications. It may strengthen societal cohesion, counter adversary narratives, and build societal resilience against hybrid threats. In an operational life cycle, EBSC should function as a Large Language Model (LLM) that senses the emotional climate of a targeted population via social-sentiment analysis, interprets this context, designs and develops EBSC messages, disseminates them through mass media, evaluates their impact, and adapts them in a real-time closed loop. EBSC thus provides a repeatable, ethically grounded framework for operating in the cognitive domain, ensuring that communications are scientifically informed, strategically consistent, and effective in reinforcing European resilience and unity.

The current contribution represents a novel avenue of application and refinement of the original concept of EBSC and dominant emotional maps, which were initially conceived by the first author nearly 20 years ago during five visits to Afghanistan as a representative of NATO Parliamentary Assembly. These concepts were explored in earlier publications in relation to extreme political attitudes, societal stress-related disorders, and processes of societal deradicalization (Cosić et al., 2011, 2012a,b, 2013, 2018). Based on these published articles as foundation, the authors made selection and integration of relevant literature to recontextualize the EBSC framework for contemporary defensive cognitive warfare.

## 2 The importance of emotion analyses in cognitive warfare

Emotions strongly influence human cognition, decision-making, and behavior (Lerner et al., 2015), particularly during and after prolonged military conflicts (Cosić et al., 2011, 2012a; Halperin et al., 2011; Halperin, 2014). Emotional arousal is not a side effect of conflict; it is often its core mechanism. The emotional contexts of war zones and war-affected societies—characterized by fear, anger, desperation, humiliation, pessimism, hopelessness, hatred, rage, frustration, and resentment—are not merely consequences of violence. They can also drive its persistence, prolong conflict, and complicate post-conflict resolution. The unchecked flow of such negative emotions increases insecurity and can make peace nearly uncontrollable.

Understanding the impact of these emotions on critical cognitive states and operational capabilities—such as situational awareness, attention, and decision-making—is therefore essential. Strong negative emotions can shape public narratives in ways that make wars more difficult to end than to prevent (Cosić et al., 2012a, 2018). Unregulated emotional escalation can render peace processes nearly impossible. When political leaders fail to recognize these dynamics, they may unintentionally reinforce emotional inertia and undermine prospects for sustainable peace. Thus, it is crucial to understand the emotional complexity of any military conflict, as these emotional forces often stand as obstacles to long-term peace.

Desire for revenge, deep anxiety resulting from violent and protracted wars, and pervasive distrust can reduce a population's receptivity to peacebuilding (Crawford, 2000). Prolonged conflict and traumatic events exert substantial influence on the emotional climate, amplifying extreme political attitudes and reigniting cycles of violence, anxiety, and distrust within the local population (Canetti et al., 2017; Hetherington and Suhay, 2011; Hall and Werner, 2022; Hong and Kang, 2017). Stress, fear, and cognitive overload impair executive functioning and diminish self-reflective capacity (Jiang and Rau, 2017; Shields et al., 2016; Arnsten, 2009), shifting responses toward reactive, symbolic, or populist tendencies rather than strategic and evidence-informed decision-making.

Without cognitive and self-reflective capacities among leaders, emotional escalation can become systemic, with media and institutional actors unintentionally reinforcing adversarial framings (Baum and Groeling, 2009; Wolfsfeld, 2004; Plowman and Walton, 2020). The increasing prominence of cognitive warfare requires reevaluating not only how military personnel are trained and supported, but also how political and institutional decision-makers understand, respond to, and shape complex emotional and perceptual environments.

Rather than replace human cognition, AI must be designed to enhance it, especially in complex operational environments where human judgment, empathy, and ethics remain irreplaceable. Cognitive assistants that support military decision-making by offering diverse perspectives, highlighting biases, and suggesting reappraisal strategies may prove extremely valuable. Integrating cognitive neuroscience and AI into such areas will require new forms of training, ethical frameworks, and institutional structures. Yet the potential benefits are substantial: a defense and security system that is not only smarter and faster, but also more adaptive, resilient, and humane.

Cognitive sovereignty refers to the ability of individuals, societies, and political systems to maintain control over their attention, judgment, emotion, and memory, even amid psychological and informational warfare. In an age of neural targeting, digital overload, and AI-accelerated persuasion, cognitive sovereignty must be treated as both a strategic resource and a fundamental human right. Protecting citizens from AI-enhanced disinformation or neuro-targeted manipulation—including technologies designed to influence emotional states in security contexts—is essential for increasing cognitive resilience. Resilience to cognitive warfare also requires a distributed capacity for emotional literacy, not only among elites but throughout democratic institutions, media and public, fostering deliberation rather than polarization, and comprehension rather than outrage.

In *Mind Wars*, Moreno (2012) raised early concerns about the potential military and national-security applications of neuroscience, highlighting the work by Defense Advanced Research Projects Agency (DARPA), and warning about technologies that could influence soldiers' memory, cognition, emotions, behavior, and mental capacities. Since the 1990s, when the U.S. National Academy of Sciences began mapping neural and anatomical brain connections, as well as exploring molecular and genetic mechanisms underlying brain function [Institute of Medicine (U.S.) Committee on a National Neural Circuitry Database, 1991; McCreight, 2024], scientific knowledge related to brain dynamics, neuronal plasticity, dendritic and synaptic interactions, neural wiring diagrams, and firing patterns associated with particular emotional or cognitive states has grown dramatically. However, this dual-use research carries risks, because neuroscience may be weaponized to degrade or impair human cognitive functions and operational neural capabilities through hostile neuromodulation (McCreight, 2024).

The EBSC approach (Cosić et al., 2012a, 2018), as presented in this article, focuses on protecting human limbic networks from hostile cognitive warfare operations that may have long-term negative impacts on reasoning and behavior. NATO's *Strategic Concept 2022* (NATO, 2022) and the EU's *Strategic Compass 2022* (EU, 2022) identify the cognitive domain as an emerging arena of contestation. Both organizations emphasize the importance of resilience, countering hybrid threats, and protecting societies against disinformation and manipulation (East StratCom Task Force, n.d.; European Digital Media Observatory, n.d.). Within this evolving politico-military landscape, EBSC offers a structured method for bridging cognitive neuroscience and AI into operational strategic communications, thereby strengthening alliance cohesion and societal resilience.

## 3 Emotionally based strategic communications

EBSC grounded in emotion recognition, affective resonance, narrative framing, and emotional influence can support defensive operations in the context of cognitive warfare. Aggregating the dominant emotions of individual members of a population in a given region of interest can capture the main sentiments and feelings perceived by participants and observers as most prominent in an episode of collective behavior. Relevant information for assessing collective sentiments and feelings can be monitored across various open digital communication sources, such as public social network channels, forums, and video-sharing platforms (Facebook, X, Reddit, YouTube, etc.), as well as portals, blogs, and news outlets.

Dominant emotional maps can be regarded as representations of the collective emotional climate and orientations of a population under consideration, providing insight into the distribution of emotions within the targeted group. These dominant emotional maps and emotional contexts can be changed or shaped through the EBSC concept, which extends and refines traditional strategic communications.

Strategic communications—aimed at influencing perceptions, attitudes, beliefs, and behaviors of a targeted audience toward a desirable end state—should explicitly integrate emotional factors, consistent with James A. Treadwell's statement: “If you want to influence someone, you have to touch their emotions” (Merle, 2005). Influencing people without engaging their emotions is almost impossible. Interpreted within a broader social and cultural environment, strategic communications that incorporate an emotionally based strategy may play a more important role in conflict management, making traditional approaches more influential and effective.

The goal of EBSC is to influence the emotions of a critical mass of members within the targeted group such that the collective emotional climate and orientation gradually shift from predominantly negative to more positive. The credibility of EBSC messages should be enhanced by focusing their narrative content on real issues and challenges on the ground and on the quality of life of the local population. Sustained delivery of EBSC messages centered on real, on-the-ground problems may contribute to the transformation of dominant emotional maps. Each successful EBSC campaign focused on a targeted population may provide an opportunity for a “tipping point” (Gladwell, 2000) in the underlying non-linear emotional dynamics, shifting the center of gravity of the dominant emotional map toward the valence–arousal quadrant associated with a positive collective emotional orientation. In defense and security contexts, EBSC are based on the design of multimodal emotional, narrative, visual, linguistic, and symbolic cues, with the objective of influencing and transforming the emotional states of the targeted population in a more positive direction.

Neurocognitive science, enhanced by AI, provides tools and methods for shaping communication strategies rooted in emotional resonance and credibility, to augment and reinforce positive public opinion and morale. Applications include counter-disinformation, deterrence, conflict de-escalation, public relations, and strengthening democratic trust.

In today's information-dense and emotionally charged strategic environment, the ability to shape affective dynamics is as critical as controlling physical terrain through kinetic power. EBSC offers a science-based framework for understanding and influencing the emotional drivers of perception, behavior, and decision-making in both military and civilian domains. Its strategic value is inherently dual-use, as it can enhance defense communications, reinforce operational capabilities and human morale, prevent radicalization, and build democratic resilience to psychological threats and cognitive warfare.

In modern hybrid and cognitive warfare environments, the ability to modulate affective dynamics is as strategically important as kinetic or cyber capabilities. The EBSC approach provides a science-based framework against adversary strategic influence campaigns by eliciting desirable emotional and behavioral responses that guide defensive decision-making processes in both military and civilian audiences, using narrative structure, language, imagery, and symbolic content. The goal is to increase the defensive robustness of emotional responses against disturbances generated by adversarial psychological operations, thereby securing internal cohesion and morale within the targeted population, as well as resilience to psychological and cognitive manipulation.

Integrating EBSC into defense strategy requires new capabilities and new AI-driven tools, such as the use of LLMs in group sentiment analysis, affective forecasting, and real-time assessment of the impact of emotionally resonant narratives on targeted populations. Combined with neuroscientific insights into affective processing, these tools can help identify which affective narratives and messages promote positive, adaptive emotional regulation, social cohesion, and threat awareness—without inciting panic or antagonism.

As such, EBSC should be embedded not only in defense communications and strategic planning, but also in civil preparedness, diplomacy, and political leadership training. Using these tools, Europe can move from reactive messaging toward emotionally informed, cognitively resilient governance. By anticipating how populations emotionally interpret ambiguous or contradictory information, EBSC leaders can design interventions that build cognitive immunity against disinformation, psychological destabilization, and societal polarization.

These methods are particularly relevant for contemporary European security, where adversaries exploit digital platforms not only to mislead, but also to manipulate emotional states in ways that undermine institutional trust. EBSC supported by AI can strengthen the cognitive mechanisms underpinning decision-making in defense and security strategic communication contexts. It may provide ontologically structured, scientifically grounded methods for designing emotionally resonant and cognitively effective narratives.

The concept of EBSC is presented in this article as a structured, scientifically grounded framework that links emotion-cognition dynamics with strategic communication in defense and security affairs. It is a conceptual and methodological framework that integrates insights from cognitive neuroscience, the psychology of emotion, and strategic communication studies to enhance the effectiveness of influence, persuasion, and resilience-building in complex defense and security environments. EBSC provides a structured, operationalizable approach that explicitly links emotional processes with cognitive mechanisms of decision-making, enabling the design of communications that are both scientifically grounded and strategically targeted.

While traditional psychological operations (PSYOPS) are military or government-planned communications intended to influence foreign audiences' emotions, attitudes, reasoning, and behavior in support of strategic objectives (Wallenius and Nilsson, 2019; Narula, 2004), EBSC places much greater emphasis on the limbic structures of the human brain. Drawing on state-of-the-art neuroscience research (LeDoux, 1996; Jost and Amodio, 2012; Salzman and Fusi, 2010; Oxley et al., 2008), EBSC seeks to design messages that emotionally resonate with targeted populations and their local cultural backgrounds, thereby making messages more impactful on human emotion, cognition, and behavior (Cosić et al., 2012a, 2018).

Emotionally anchored narratives can circumvent purely rational resistance, as decisions under conditions of stress or threat are often made emotionally first. This makes EBSC a powerful amplifier in defensive disinformation campaigns, deterrence messaging, and alliance-building. EBSC can be paired with AI-driven sentiment analysis and neurocognitive models to predict how targeted populations exposed to aggressive cognitive warfare might respond to specific triggers. It can enhance defensive emotional regulation within groups, creating shared positive emotional states that counter fear, radicalization, and distrust.

By steering emotional climates, EBSC can reinforce alliance cohesion, strengthen resilience against adversary influence, and inoculate populations with emotionally resonant counter-narratives that build trust in institutions. Finally, EBSC offers a structured, transparent, and ethical approach in democratic contexts, enhancing trust, promoting unity, and reducing susceptibility to hostile influence.

## 4 Transforming dominant emotional maps through emotionally based strategic communications

Narrative engineering based on generative AI (DeBuse, 2024; Chan, 2025a,b) will be capable of constructing and delivering messages that resonate emotionally while guiding cognitive interpretations toward desired political, defense, and security outcomes. EBSC enables strategic transitions from reactive emotional states to cooperative, resilient states through deliberate narrative design. This is particularly critical for countering adversarial influence campaigns that exploit emotional vulnerabilities through misinformation and polarization.

The evolving nature of modern warfare and security challenges demands a transformation in how defense capabilities are conceived, developed, and deployed. EBSC provides tools for identifying and transforming dominant emotional states. Through intentional narrative design, communicators can support transitions from reactive, fear-based cognition to reflective and cooperative engagement. This is highly relevant in hybrid warfare environments, where adversaries aim to destabilize democratic societies by manipulating emotional vulnerabilities via disinformation and polarizing media campaigns.

EBSC leverages structured multimodal narratives—integrating language, imagery, and symbolic framing—to positively reconfigure collective emotional states without coercion. By incorporating neuroscience-informed insights into cognitive performance, perception, stress, and attention, and by combining these with adaptive and neuro-symbolic AI systems, EBSC enables the development of more robust, responsive, and ethically grounded defense solutions. This convergence may also contribute to the formation of a new European strategic decision-making ecosystem, one that leverages real-time monitoring of the targeted population's cognitive and emotional states, supported by AI and EBSC.

This article proposes a conceptual framework to guide policy research and outlines implications for European strategic autonomy, ethical governance, and cross-disciplinary innovation. While cognitive performance in operational settings is critical, the broader strategic environment increasingly hinges on emotional dynamics at societal and political levels. In an age of information saturation and psychological manipulation, EBSC emerges as a core tool for defending democratic stability and managing the emotional architecture of collective decision-making.

Emotional management in the form of EBSC as “soft power” is essential for stabilizing war-affected societies (Cosić et al., 2012a,b, 2018). Assessing dominant emotional patterns among the populations of these societies is crucial for achieving and restoring peace, stability, and security. Once local dominant emotional maps, meanings, and interpretations are identified and understood, influencing the behavior of the local population becomes possible through a comprehensive EBSC policy.

**Figure 1:**
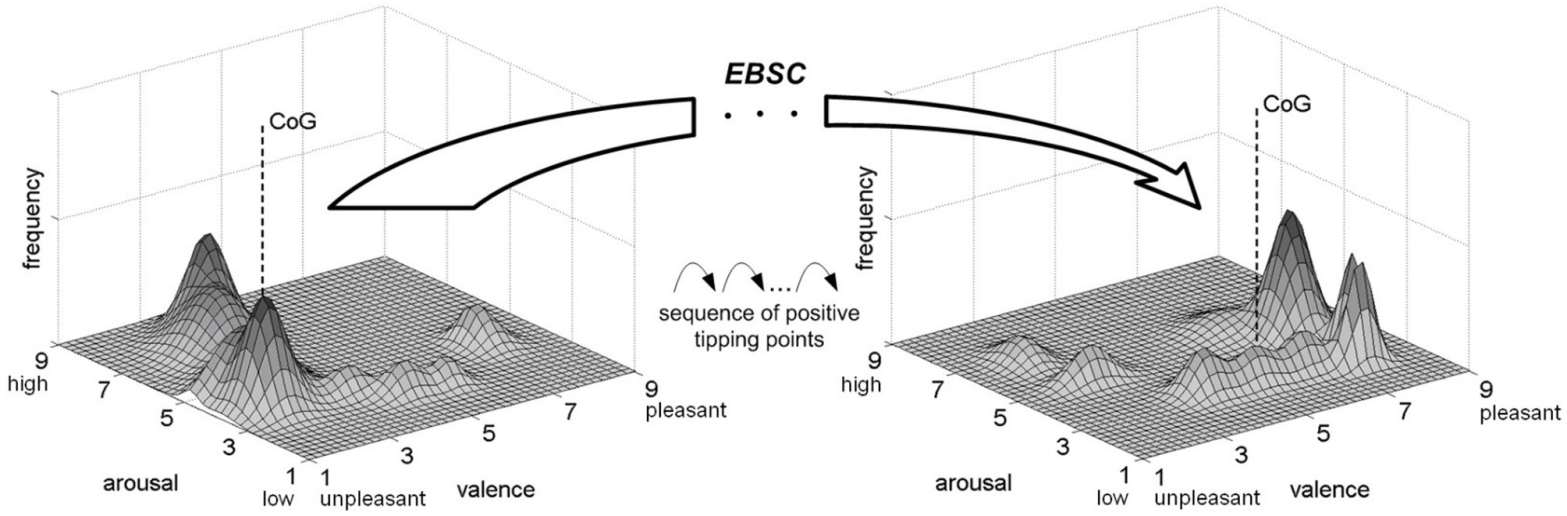
Transformation of dominant emotional maps by EBSC (Cosić et al., 2011, 2012a).

The transformation of a negative dominant emotional map in a war-affected society into a desirable positive dominant emotional map—facilitated by EBSC—is illustrated in Figure 1. Dominant emotional maps within the framework of EBSC are structurally embedded, collectively shared cognitive-affective statistical visualizations that determine how target audiences emotionally frame strategic war reality, thereby shaping perception, meaning attribution, legitimacy judgments, and decision predispositions prior to and during exposure to strategic communication. They are displayed as histograms showing the distribution of emotions experienced by all individuals in a society or a group of interest at a particular time, enabling visual identification of the most frequently experienced, dominant emotions. The negative dominant emotional map of the war-affected society, illustrated in the left part of Figure 1, is characterized by the predominance of trauma-induced negative discrete emotions such as fear, anger, humiliation, sadness, and frustration (Ekman, 1992; Roseman, 1991; Demszky et al., 2020), which occupy unpleasant/high-arousal region in the valence–arousal space of dimensional emotion models (Russell, 1980; Barrett, 1998; Posner et al., 2005; Buechel and Hahn, 2017). Physiological and cognitive-behavioral signatures associated with these emotions include activation of hypothalamic-pituitary-adrenal axis, structural remodeling in the prefrontal cortex, heightened aggression, reduced pro-social behavior, susceptibility to misinformation and even violent extremism (McEwen and Morrison, 2013; Cohen, 1980; Martel et al., 2020; Mohammed and Neuner, 2022; Lewis et al., 2024).

The ultimate goal is to shift the emotions of a critical mass of individuals within the war-affected society so that, through a series of tipping points, the dominant emotional map begins converging toward the desirable end state, illustrated by the map in the right part of Figure 1. This end state is characterized by positive emotions dominating in dimensional valence–arousal space and discrete emotion space (hope, optimism, satisfaction, calmness, etc.), as well as by cognitive-behavioral signatures such as critical thinking and fact-checking, productive citizenship, adherence to social order and norms, and support for other pro-social values. For example, peacebuilding radio drama communications in post-genocide Rwanda were shown to shift perceived social norms and behaviors relevant to intergroup tolerance and reconciliation, increasing willingness to express independent thought, as well as impacting constructive communal problem-solving and dispute resolution (Paluck and Green, 2009; Paluck, 2009).

Transformation of the dominant emotional map through EBSC (Figure 1) should result in a controllable trajectory of the map's Center of Gravity (CoG)—representing the average valence–arousal values of individual emotions in the targeted group or society—moving from the unpleasant, high-arousal region toward the pleasant, lower-arousal region of the valence–arousal space.

Successful transformation of dominant emotional maps depends on coordinated efforts among political decision-makers, the international community, and the transformational potential of the entire society or specific target groups within highly fragmented societies. It is important to emphasize that EBSC is not a panacea for all societal problems exploited and amplified by adversarial cognitive warfare propaganda. Its chances of success are limited if implementation efforts are not accompanied by simultaneous economic, political, security, and institution-building measures. Only when embedded within these broader processes can strategic emotional management via EBSC contribute meaningfully to societal transformation.

The objective of EBSC is to deliver semantically and emotionally appropriate multimedia stimuli, drawn from specifically designed emotional databases, to a targeted group or nation experiencing cognitive warfare aggression. Based on comparative analysis between the delivered semantically and emotionally annotated stimuli and their impact on the dominant emotional map of the targeted group, an adaptive emotional expert system supported by an LLM updates the stimulation strategy to strengthen the group's resilience against adversarial cognitive warfare. EBSC messages are cognitively interpreted by members of the targeted group within their specific historical, cultural, and political context.

The integration of emotional properties and narrative content within LLM-based EBSC must be designed with enough flexibility to emphasize the right issues at the right time (Corman and Dooley, 2008)—strongly, effectively, and with full confidence and credibility—serving, for example, as inoculation against imposed cognitive warfare. The most successful combinations of emotional properties and narrative content are those that embrace ideas and emotions capable of quickly gaining resonance with the targeted group. Narrative content should focus on real achievements, functional institutions, and credible economic results that are likely to be judged positively by the targeted group rather than perceived as controversial or negative.

However, public opinion is shaped not only by words but also by emotionally grounded perceptions. Therefore, when conveying information, it is essential to consider the culture, history, and traditions of intended audiences. Understanding and accounting for specific psychosocial mental complexes, socio-cultural and socio-political structures, and their interdependencies is crucial for designing effective EBSC messages directed at specific audiences. Relevant literature demonstrates gender- and age-related differences in self-estimated cognitive-emotional capacities and related psychological correlates (Giannouli, 2023), as well as emotional appraisal processes (Stella et al., 2022), underscoring the need for EBSC approaches to account for demographic and cross-cultural diversity rather than assuming homogeneous audience responses.

EBSC can be regarded as a “soft approach” to winning the hearts and minds of a targeted population, strengthening their reluctance to endorse hostile narratives and reinforcing their resistance to cognitive warfare directed against their emotional brains (LeDoux, 1996). In other words, EBSC can be viewed as a comprehensive process that produces and shapes “soft power” (Nye, 1990), offering a cost-effective complement to currently predominant “hard power” approaches. As such, EBSC may provide real added value as a form of soft power aimed at guiding a targeted population toward a more positive end state.

A well-designed EBSC approach—implemented through credible and transparent public campaigns—can induce vitally important positive emotions and a sense of efficacy, cultivating a genuine positive outlook among the targeted population while maintaining appropriate public vigilance during conflict situations (e.g., Lewandowsky and van der Linden, 2021; Ecker et al., 2022).

## 5 Emotionally based strategic communications as an LLM closed-loop transformer

The conceptual flow illustrating how EBSC should be embedded into cognitive warfare involves several stages: AI-based analysis of data to identify emotional and cognitive vulnerabilities; an EBSC transformer that translates these insights into emotionally precise influence strategies; narrative design that crafts and deploys strategic messaging; and a target audience that receives these messages and reacts at cognitive, emotional, and behavioral levels. Throughout this process, feedback loops measure resonance and adapt communications in real time. This creates a closed-loop system for the continuous optimization of influence in cognitive warfare, defending against adversary narratives by understanding how emotions shape cognition, behavior, and decision-making.

In defensive cognitive warfare, EBSC functions as a neuroscientifically grounded framework for narrative engineering (DeBuse, 2024), countering offensive narratives through positive, fact-based, emotion-adapted messaging. The convergence of cognitive neuroscience and AI embedded within EBSC may create a novel pathway for Europe to build adaptive, human-aligned, and ethically governed defense capabilities against offensive cognitive warfare. Embedding EBSC within defense training and education, public diplomacy, and crisis response ecosystems offers proactive tools for shaping emotionally stable strategic landscapes.

Real-time feedback loops based on emotion and sentiment analysis continuously and dynamically refine EBSC strategic messaging in a closed loop for optimal defensive impact. LLM-selected multimedia stimuli can be broadcast by radio, leaflets, television, and other traditional as well as digital dissemination channels, whose number continues to grow. In societies with low levels of technological development, however, mass media broadcasting alone cannot be assumed sufficient to induce positive change and must be complemented by person-to-person dissemination of messages at formal and informal meetings and other public gatherings.

EBSC as an LLM transformer can be interpreted, in the context of closed-loop systems theory, as a regulation—similar to a proportional-integrative-derivative (PID) controller in a closed-loop system—whose objective is to improve system performance in terms of stability, accuracy, and overall effectiveness. In the context of this article, regulation is related to mental states of targeted population such as cognition, emotion, and behavior, which are disturbed by adversarial kinetic or cognitive warfare aggression. Under dangerous, life-threatening circumstances, individuals may shift from a statistically normal distribution of emotions to a dominant emotional map characterized by fear, sadness, anger, and related states, which can contribute to depression, anxiety, PTSD, or even suicide.

Using a well-trained EBSC LLM transformer, emotionally charged inputs—messages, sentiments, narratives, etc.—can be systematically transformed into strategically framed output messages with the goal of preventing further degradation of the dominant emotional map of a targeted population. Emotional input mapping is based on collecting emotional content such as messages, texts, posts, speeches, and interviews. By applying emotion and sentiment analysis methods, the EBSC input layer classifies emotions (e.g., fear, anger, hope, grief) and places the results into a “dynamic emotional map” that models the targeted community's emotional state distribution over time and context.

The transformation methodology is grounded in these emotional inputs, which are mapped, processed, and reframed positively to support resilience, empathy, and stability in communications with the targeted population. Rather than amplifying volatility, the EBSC process should acknowledge and validate underlying emotions that people need to have recognized. Reframing requires shifting negative valence into constructive orientations—for example, fear into preparedness, anger into justice-seeking. It is important to avoid emotional overload by blending calming, hopeful, and practical tones, and by linking output narratives to shared values, identity, or positive collective memory. The following subsections outline how emotional input can be mapped, processed, and reframed constructively to support resilience, empathy, and stability in communications.

### 5.1 LLM-based EBSC transformer as a three-stage filter

**Figure 2:**
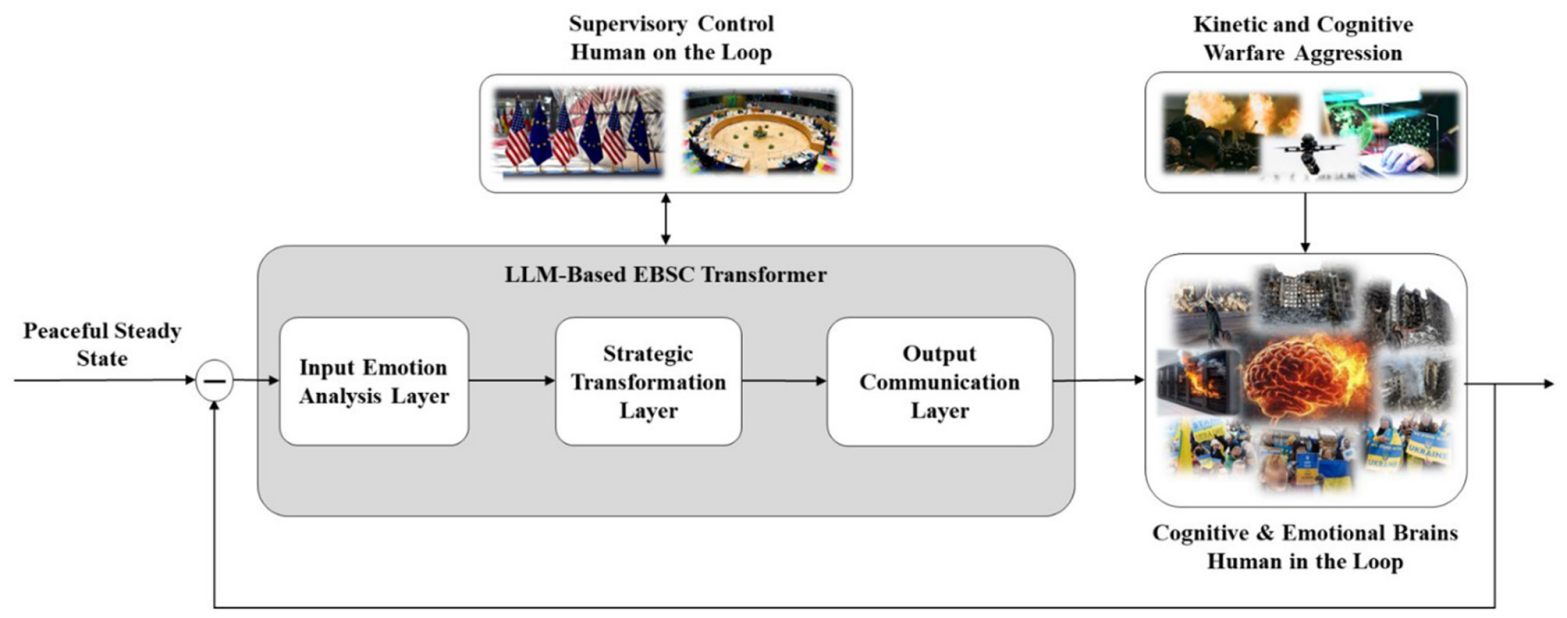
Cognitive warfare defense strategy based on cognitive neuroscience, AI and EBSC.

The LLM-Based EBSC Transformer can be viewed as a three-stage filter composed of three layers (Figure 2).

#### 5.1.1 Input emotion analysis layer

This layer extracts keywords, emotional tendencies, intensity, and potential triggers in near real time (hourly or daily). It collects emotional messages (texts, posts, speeches, interviews) in natural language form from diverse open digital communication channels and sources such as public social networks (Facebook, X, etc.), forums (Reddit, Discord, etc.), video-sharing sites (YouTube, TikTok, etc.), Telegram channels, civic portals, blogs (e.g., WordPress), news sites, and broadcast transcripts. Sentiment analysis and emotion classification (Hasan et al., 2019; Rosado et al., 2022) are applied to generate a dynamic emotional map (Cosić et al., 2012a,b)—a model of the community's emotional state distribution over time and topics, supplemented with geographic location.

This layer also tracks keywords; context/topic categories (e.g., energy, sanctions); narrative clusters within these categories (e.g., “energy costs are rising,” “intensify sanctions”); classified emotions according to valence–arousal or discrete-emotion models (e.g., joy, fear, anger, hope, grief); identified positive and negative sentiments; frames (Wang et al., 2025) as interpretive lenses that give narratives their meaning (e.g., threat, injustice, betrayal, dignity, efficacy, fairness); and supporting or opposing stances (Küçük and Can, 2020) toward specific institutions, actors, policies, themes, claims, or propositions. It further assigns cognitive warfare–related labels such as bot likelihood (Yang et al., 2022), coordination signals (Cinelli et al., 2022), message toxicity (Lees et al., 2022), and misinformation content (Islam et al., 2020), and computes confidence/uncertainty estimates for these inferred constructs to facilitate decision-makers' situational awareness.

#### 5.1.2 Strategic transformation layer

This layer maps emotional inputs into constructive outputs—for example, transforming fear-related wording into messages centered on preparedness, safety, and encouragement; anger into productive and fruitful action; sadness into solidarity; erosion of trust into hope; and joy into reinforcement and strength. Its primary focus is to validate underlying emotions, shift negative valence toward constructive orientations, and avoid emotional overload by incorporating calming, hopeful, and practical tones.

Through cyber text/speech and social sentiment analysis across EU/NATO audiences, this layer translates cognitive and affective inputs into output narrative design and strategy, while continuous assessment of effects drives real-time adaptation. Mechanisms in this layer include diversifying emotional usage (avoiding overreliance on fear or outrage); connecting narratives to shared values, identity, or positive collective memory, culture, and habits of the targeted population; and strategically injecting positive emotions such as hope, pride, and gratitude to stabilize negative dominant emotional maps.

Transforming emotional inputs into EBSC outputs involves cognitive reappraisal, reframing, and redirection while preserving emotional diversity, avoiding the dominance of destructive emotions, and maintaining social peace and stability, thereby crafting a more positive collective emotional climate. The strategic transformation layer receives information from input emotion analysis layer and analyzes dominant emotional maps, sentiments, cognitive biases, broader defense and security threats, and relevant social and environmental context.

This layer may also support mental preparedness and readiness in the targeted population by building resilience, inoculating against disinformation, and reinforcing trust in institutions. It can additionally contribute to mental health support (Cosic et al., 2024) by using the wording, language and principles of computerized cognitive-behavioral therapy (Vallury et al., 2015), stress inoculation training (Wiederhold and Wiederhold, 2010; Meichenbaum, 2017), stress exposure training (Driskell et al., 2018), mental readiness training (Thompson and McCreary, 2006), emotion regulation frameworks (Braunstein et al., 2017; Renna et al., 2017), cognitive restructuring (Clark, 2013), and mindfulness training (Baer, 2003; Stanley, 2014). Technology-supported training and monitoring can function as a form of ‘stress vaccine,' strengthening adaptive coping responses before exposure to high-stress environments (Wiederhold, 2024a).

To respond in real time to the evolving dynamic emotional map of a targeted population (on an hourly or daily basis), the LLM-Based EBSC Transformer is expected to operate partially autonomously under human supervision—for example, at higher levels of the 10-level taxonomy proposed by Sheridan and Verplank (1978). Balancing real-time responsiveness with mandatory human oversight suggests the need to allow partially automated crafting and transmission of multimedia messages, while keeping human supervisors in the loop. In more extreme cases, where dynamic emotional maps exhibit profound volatility and negative collective orientations, strategic transformation layer can alert human supervisors to collaborate on message crafting and approve the transmission of messages to stabilize these highly volatile emotional maps.

Within the closed loop, this layer augments human decision-makers' cognitive awareness and metacognitive self-awareness, as well as their cognitive clarity, literacy, and flexibility, thereby increasing cognitive dominance and operational preparedness and readiness. It also mitigates lapses due to cognitive biases and supports more unbiased decision-making under stress and uncertainty, enabling effective responses in crisis situations and facilitating decision-making superiority. This layer broadens and enhances perception, cognition, working memory, attention, situational awareness, and decision-making performance, countering the detrimental effects of fatigue, strong emotions, and cognitive overload. It supports emotional regulation and control over attention, judgment, emotion, and memory; helps to manage stress, bias, and uncertainty; and contributes to stress resilience and mental health in inherently high-stress decision-making environments. Altogether, it supports human judgment, real-time analytical capacity and endurance, foresight and risk calibration, integrity, empathy, and ethics—promoting responsible, anticipatory rather than reactive governance and leadership, and reducing reliance on fast, inadequately thought-through heuristic decisions in high-stakes, emotion-laden environments.

LLM-driven multilingual discourse analysis and predictive models of influence, in the face of adversarial pressure, can also enable tracking of cognitive changes related to attention, memory, and resilience factors among targeted populations.

#### 5.1.3 Output communication layer

This layer distributes and transmits multimedia messages that resonate emotionally, constructively guide audiences toward stabilizing narratives, and remain coherent with the broader communication strategy. It includes digital and traditional channels such as social media, online platforms, leaflets, radio, television, newspapers, and public gatherings.

Supervisory control by humans “on the loop” (van Diggelen et al., 2025) of proposed closed-loop system should be conducted in line with appropriate, well-founded EU/NATO ontologies and methodologies (Bebber, 2025). Understanding the local values, culture, habits, emotions, and sentiments of the targeted population is critically important as part of the necessary personalization of EBSC to achieve meaningful performance and efficiency.

EBSC serves as the translation engine, turning cognitive neuroscience and AI insights into legitimate, emotionally resonant, and strategically aligned communications that reinforce EU/NATO cohesion, counter and outpace adversary narratives, and strengthen societal resilience. Accordingly, the EBSC approach goes beyond “quick technological fixes” against offensive cognitive warfare—such as “AI-based early warning systems” or “state-run, internet-wide content moderation tools”—which have been criticized in the literature (Lahmann et al., 2025). Instead, LLM-based AI tools with deep cognitive understanding of the multidimensional and complex nature of targeted populations—psychologically, politically, economically, and in security terms—are used within EBSC to extend situational awareness (Endsley, 1995, 2000), and increase the effectiveness of observe–orient–decide–act (OODA) loops (Boyd, 1996; Ho and Kuah, 2014; Tanui, 2021; Daniels, 2021). Decision-makers, as supervisory humans “on the loop,” must retain ultimate accountability for the strategic communications messages disseminated to targeted audiences. The emerging discourse on humane AI underscores that defense-related AI systems should be designed around human dignity, transparency, and proportionality—principles consistent with EBSC's emphasis on ethically grounded, democratically accountable influence operations (Riva et al., 2025).

Such requirements align with similar considerations in prior literature (van Diggelen et al., 2025; Taddeo and Floridi, 2018b) and with auditing proposals for large-scale AI systems (Raji et al., 2020), particularly those involved in opinion dynamics control (DeBuse and Warnick, 2023, 2024). Likewise, the LLM-based EBSC Transformer proposed in this article should ensure compliance with International Humanitarian Law (Rodenhäuser, 2023), the EU AI Act, the General Data Protection Regulation (GDPR), the Tallinn Manual 2.0 on the International Law Applicable to Cyber Operations (Schmitt, 2017), and other applicable EU regulations.

## 6 Enhancement of European defense policy by emotionally based strategic communications

The proposed closed-loop LLM-based EBSC Transformer could be integrated into European defense strategic communications policy and aligned with EU frameworks on resilience and counter-disinformation (Delwaulle, 2024; European Commission, 2018; Rød et al., 2025). At the recent panel at the Dubrovnik Forum (11–12 July 2025), titled “European Defense Policy Enhanced by Convergence of Cognitive Neuroscience and Artificial Intelligence” (Government of Croatia, 2025; MVEPRH, 2025), participants discussed the potential of merging cognitive neuroscience and AI to bolster EU defense capabilities during military stressful operations.

In the introductory remarks, it was emphasized that “in the evolving domain of European defense and security affairs, cognitive neuroscience and AI should emerge as influential forces which can significantly enhance European defense potential when global threats arise rapidly, and when Europe faces a pressing need to enhance its defensive cognitive warfare strategies, leveraging theoretical and applied knowledge from cognitive neuroscience and AI. Having interdisciplinary knowledge to identify relevant features and develop AI-based models for prediction of potential military aggression early enough is extremely important. Therefore, AI-based cognitive superiority within the European defense system should be extremely valuable as strategic guidance for future work on a complex, distributed, and multi-layered AI-based defense system, laying the foundation for our future joint research on European defense, security, and operational superiority.” This message reflects and builds upon similar previous calls for enhanced use of AI for European defense in the area of cybersecurity (Taddeo and Floridi, 2018a).

Cognitive neuroscience can support a deeper understanding of human emotion, cognition, and behavior in defense strategic contexts, providing innovative insights into perception, beliefs, trust, situational awareness, decision-making, attention, leadership, working memory, mental stress, stress resilience, mental health, operational readiness, fatigue, and cognitive overload as key operational defense capabilities (Cosić et al., 2022).

Neuroscience also reveals how military leaders manage stress, bias, uncertainty, and complex warfare scenarios, enabling risk-informed strategic foresight. It shows how repeated exposure to propaganda shapes beliefs (Hebb's rule, “fire together, wire together”). Cognitive warfare targets not only soldiers, but entire societies, undermining trust in institutions and public perception that can induce societal polarization and destabilize democratic systems. AI systems could analyze how adversarial disinformation spreads across European media and social networks (Pacheco et al., 2021; Cinelli et al., 2022; Sharma et al., 2021; Marigliano et al., 2024), allowing European defense and security policymakers to respond faster and more effectively. Recent work in cyberpsychology similarly emphasizes that countering AI-driven disinformation requires an integrated approach combining technical detection, strategic communications, and psychological resilience-building at the societal level (Wiederhold, 2024b).

However, neither cognitive neuroscience nor AI can replace sound human democratic political judgment. Working together, they can amplify human reasoning, sharpen situational awareness, and enhance strategic depth. Integrating cognitive neuroscience and AI into European defense and security affairs promises more adaptive, resilient, and effective strategies that address the complexities of modern threats. This interdisciplinary approach has the potential to enhance operational capabilities, improve effectiveness, strengthen mental resilience and wellbeing among soldiers, improve mission outcomes, and ultimately ensure greater security and stability. It also promotes a deeper understanding of human factors such as cognitive limitations, overload, and fatigue, which can undermine defense performance and capabilities.

AI can analyze vast amounts of data to identify patterns and predict potential threats, while cognitive neuroscience can provide insights into how decision-makers think and react under pressure (Porcelli and Delgado, 2017; Arnsten, 2009). This synergy can lead to improved situational awareness and more effective responses in crisis situations. Cognitive augmentation, real-time analytical capacity, and predictive simulation in support of decision-making under uncertain, complex conflict and negotiation scenarios—based on AI—can enhance tactical, operational, and strategic speed through predictive capabilities.

The interdisciplinary approach combining cognitive neuroscience and AI holds great promise for enhancing our understanding of strategic decision-making and creating more sophisticated predictive models that can be applied across various sectors to develop more adaptive, resilient, and effective strategies for modern and future warfare operations. Europe's strategic future will not be determined solely by kinetic force, but increasingly by cognitive readiness (Fletcher and Wind, 2013; Grier, 2012), adaptive cognitive neuroscience and AI integration, and strategic, emotionally based multimodal communications. These issues should not remain a theoretical ambition, but should be treated as a strategic necessity, forming the foundation of a new generation of defense capabilities centered not only on precise firepower, but on the development of predictive models that function as powerful preventive and proactive tools in European strategic defense and security affairs.

EBSC supported by cognitive neuroscience and AI offers tools to identify, track, and deconstruct emotional trajectories in real time in turbulent warfare environments and contexts of societal instability. For example, sentiment analysis of political discourse can detect shifts in public mood and policy framing, while AI-supported decision dashboards can be designed to reduce bias and highlight overlooked information. These tools, however, are only effective if decision-makers are trained in cognitive awareness and supported by systems that reward strategic patience over emotional reflexes. This may be especially challenging for “dark leadership” decision-maker profiles (Giannouli, 2025), as hubristic overconfidence and narcissistic status sensitivity can bias threat appraisal, reduce receptivity to corrective feedback, and incentivize the strategic use of outrage or humiliation rather than emotional regulation (Owen and Davidson, 2009; Asad and Sadler-Smith, 2020; O'Reilly and Hall, 2021; Gauglitz et al., 2023), particularly when evidence from AI-supported decision dashboards is not aligned with leaders' entrenched beliefs or self-image. Similarly, at the group level, collective narcissistic beliefs are associated with heightened threat sensitivity and hostility (Golec de Zavala, 2019; Hase et al., 2021), and the amplification of these reactions via emotional contagion (Barsade, 2002) may drive the group's pushback against de-escalatory strategic communications.

When EBSC is embedded in leadership training and strategic planning, it can help political actors navigate emotionally volatile environments without reinforcing cycles of escalation. EBSC enables a shift from emotional contagion to emotional regulation, and from reactive politics to anticipatory governance. Comparable multilevel emotion-cognition dynamics have also been documented in non-politico-military settings, such as emotionally demanding workplace environments, where leaders' emotional regulation, leadership styles, and affective signaling shape subordinate cognition and decision-making, with implications for organizational resilience (Giannouli, 2017; Bataineh et al., 2025). Crucially, resilience to cognitive warfare requires a distributed capacity for emotional literacy, not only among elites but throughout democratic institutions. Public education, media literacy, and institutional design must align to foster deliberation rather than polarization, and comprehension rather than outrage. Europe's long-term security depends not only on technological advancement, but also on the emotional and cognitive sophistication of its political culture. Experience from multinational peacekeeping training initiatives indicates that structured readiness and resilience programs are strongly valued and can be aligned with broader NATO/EU goals in human-centric defense (Wiederhold and Wiederhold, 2024). Similarly, proposals to integrate cognitive-neuroscience-informed decision support with AI-enabled situational awareness highlight how human-centric, ethically governed technologies can enhance mission effectiveness and civilian protection (Wiederhold, 2025a).

The history of conflict has shown how information warfare—including disinformation, social media manipulation, and, more recently, deepfakes—can destabilize societies. Integration of neuroscience and AI can support the detection of cognitive vulnerabilities exploited by hostile actors (e.g., identity threats, fear contagion), and can contribute to the design of resilient information environments that reinforce cognitive clarity and emotional regulation. This may be facilitated by transparent, credible, and responsible systems for monitoring psychological stress across populations, enabling early interventions to prevent societal destabilization.

Complex EBSC layers enable affective framing that reinforces cohesion, builds trust and solidarity, and strengthens resilience by crafting counter-narratives in line with EU/NATO deterrence and defense postures to neutralize hostile influence. A closed-loop operational lifecycle should enable the collection and fusion of open-source/social media data and protected mission-specific signals and data, consistent with EU/NATO practices, generating cognitive-emotional battlespace maps that identify vulnerabilities and influence pathways.

AI-based design of EBSC messages should translate such models into emotionally resonant strategic communications that are affective, lawful, and consistent with EU/NATO narratives across official EU/NATO channels, partner states and governments, and digital media platforms. The strategic value of EBSC lies in its contribution to EU/NATO cohesion, through emotionally resonant communications that strengthen trust and solidarity among allies and prepare publics to resist adversarial disinformation and psychological pressure. EBSC doctrinal innovation that bridges neuroscience, AI, and communication into a new European strategic decision-making ecosystem may enhance EU defense and security policy and increase EU operational effectiveness, ensuring that NATO and the EU would maintain an edge in the cognitive warfare domain.

EBSC aims to protect, harden, and uplift public cognition, building societal and institutional integration, trust, cohesion, and agency based on LLM-assisted content analysis and synthesis. EBSC narratives should reinforce resilience through inoculation, pre-bunking, sense-making support, and pro-social framing (Lewandowsky and van der Linden, 2021; Roozenbeek and van der Linden, 2021; van Diggelen et al., 2025; Reuter et al., 2025; Defazio et al., 2021; Banker and Park, 2020). These align closely with inoculation-based approaches that expose individuals to weakened forms of manipulation to build psychological resistance against future disinformation attempts (Wiederhold, 2025b).

A range of computational tools can be applied within the EBSC framework, including automated emotion detection and sentiment analysis (Nandwani and Verma, 2021; Hasan et al., 2019; Mohammad, 2016; Rosado et al., 2022); narrative extraction and synthesis (Santana et al., 2023; Overney et al., 2025; Yan and Tang, 2023; Keith Norambuena and Mitra, 2021); causality and relations extraction (Ali et al., 2023; Zhao et al., 2024); automated claim detection (Guo et al., 2022; Choi and Ferrara, 2024; Sheikhi et al., 2023); and automated stance detection (Küçük and Can, 2020; Li et al., 2021; Garg and Caragea, 2024; Hardalov et al., 2022). Additional tools include social botnet detection (Zhang et al., 2018; Patil and Deshpande, 2022), credibility assessment in social networks (Alrubaian et al., 2017a,b; Viviani and Pasi, 2017), social media anomaly and emergency detection (Yu et al., 2016; Huang et al., 2022), and privacy-preserving data analysis (Dwork and Roth, 2014; Huang et al., 2020).

Furthermore, EBSC complements other approaches for strengthening resilience against cognitive warfare, such as media literacy and pre-bunking curricula that teach recognition of common manipulation tactics (Lewandowsky and van der Linden, 2021; Traberg et al., 2023); instructional design using spaced practice to improve retention of skills related to recognizing manipulation cues (Cepeda et al., 2006, 2008); and rumor-control resources that centralize clarifications and reduce uncertainty during cognitive warfare incidents (European Digital Media Observatory, n.d.; Innovative Data Intelligence Research Laboratory, n.d.; Full Fact, n.d.).

It will also be important to define and track appropriate key performance indicators to monitor the progress of EBSC implementation and execution, as well as the effectiveness of EBSC in terms of its intended impact on targeted populations. These indicators represent practical measures of effectiveness that are essential for evaluating and refining EBSC in operational contexts.

## 7 Conclusion

EBSC may serve as the transformation and translation engine, turning cognitive neuroscience and AI insights into legitimate, affective, emotionally resonant, and strategically aligned communications that reinforce EU/NATO defense cohesion, counter adversarial narratives, and strengthen societal resilience. In this way, EBSC contributes to the emerging European strategic decision-making ecosystem, offering a scientifically robust and ethically adaptable tool for protecting human perceptions, attitudes, and collective behaviors in the cognitive domain.

The potential of EBSC in the context of European defensive cognitive warfare lies in its capacity to enhance the emotional and cognitive capabilities of individuals and groups, thereby amplifying the effectiveness of societal defense. Grounded in a deep cognitive understanding of the multidimensional and complex human psychological, political, and economic dimensions of security, the proposed LLM-based EBSC approach can strengthen human cognitive, emotional, and behavioral processing capacities in defensive cognitive warfare, particularly in the battle for hearts and minds of targeted populations.

Its real power lies in coupling scientific models of emotion-cognition interaction with AI-enabled narrative delivery systems to respond effectively to cognitive warfare operations directed toward European societies by any current or future adversaries.
